# Genetic characterization of Addison’s disease in Bearded Collies

**DOI:** 10.1186/s12864-020-07243-0

**Published:** 2020-11-26

**Authors:** Liza C. Gershony, Janelle M. Belanger, Marjo K. Hytönen, Hannes Lohi, Thomas R. Famula, Anita M. Oberbauer

**Affiliations:** 1 Department of Animal Science, University of California-Davis, Davis, CA 95616 USA; 2 Brazilian National Council for Scientific and Technological Development (CNPq) fellow, Brasilia, DF 71605 Brazil; 3 Department of Medical and Clinical Genetics, and Department of Veterinary Biosciences, University of Helsinki, 00014 Helsinki, Finland; Folkhälsan Research Center, Helsinki, 00290 Finland

**Keywords:** Addison’s disease, Autoimmune, Dogs, GWAS, Genomics, Hypoadrenocorticism

## Abstract

**Background:**

Primary hypoadrenocorticism (or Addison’s disease, AD) is an autoimmune disease that results in destruction of the adrenal cortex and consequent adrenal insufficiency. The disease has been described in purebred and mixed breed dogs, although some breeds, including the Bearded Collie, are at increased risk for AD. Candidate gene approaches have yielded few associations that appear to be breed-specific. A single other genome-wide association study reported no significant regions of association for AD in Standard Poodles. The present study aimed to identify genomic regions of association for canine AD in Bearded Collies.

**Results:**

Our study consists of the first genome-wide association analysis to identify a genome-wide significant region of association with canine AD (CFA18). Peaks of suggestive association were also noted on chromosomes 11, 16 and 29. Logistic regression analysis supported an additive effect of risk genotypes at these smaller effect loci on the probability of disease associated with carrying a risk genotype on CFA18. Potential candidate genes involved in adrenal steroidogenesis, regulation of immune responses and/or inflammation were identified within the associated regions of chromosomes 11 and 16. The gene-poor regions of chromosomes 18 and 29 may, however, harbor regulatory sequences that can modulate gene expression and contribute to disease susceptibility.

**Conclusion:**

Our findings support the polygenic and complex nature of canine AD and identified a strongly associated locus on CFA18 that, when combined with three other smaller effect loci, was predictive of disease. The results offer progress in the identification of susceptibility loci for canine AD in the Bearded Collie. Further studies are needed to confirm association with the suggested candidate genes and identify actual causative mutations involved with AD susceptibility in this breed.

## Background

Primary hypoadrenocorticism or Addison’s disease (AD; OMIA 000520–9615) results from immune-mediated destruction of the adrenal cortex, leading to a life-threatening clinical condition characterized by inadequate secretion of adrenocortical hormones. The disease has been reported in many purebred and mixed breed dogs [1, 2], with prevalence up to 0.5% in the overall canine population [3–7]. However, multiple breeds appear to be predisposed to AD [2, 8–10], including Bearded Collies, Portuguese Water Dogs (PWD), Standard Poodles, West Highland White Terriers, Leonbergers, Wheaten Terriers, and Nova Scotia Duck Tolling Retrievers (NSDTRs) [2, 5, 7, 11–13], and disease prevalence in these breeds can be as high as 9% [4, 14–16].

Similar to humans, studies on canine AD have suggested a female predisposition [7, 12], although males and females appear to be equally affected in Bearded Collies [16], PWD [1] and Standard Poodles [15]. Average age of onset is 4 years for all dog breeds [2, 7, 11], and most dogs are diagnosed between 2 and 7 years of age [17]. An exception is the NSDTR, for which average age of onset is 2 to 3 years [7, 13]. However, recent findings suggest that the NSDTRs are affected by two types of AD: a juvenile type often seen as part of a multisystemic autoimmune syndrome and diagnosed at a much earlier age, and an adult-onset AD that corresponds to the AD seen in other dog breeds [18].

Disease pathogenesis is similar in dogs and humans [2, 8–10, 19], with lymphocytic infiltration of adrenal tissue, autoantibody production against adrenocortical antigens and impaired steroidogenesis characterizing AD in both species [19–22]. Although autoantibodies against adrenal antigens are observed in > 80% of human AD patients [21], providing important diagnostic and predictive information, in dogs, autoantibodies have only been identified in 24% of AD patients [23], indicating limited usefulness as a diagnostic tool for canine AD.

Genetic predisposition for disease is evident in both dogs and humans [1, 8, 15, 16, 19, 24, 25], and genes implicated in increased susceptibility to human AD include the major histocompatibility complex (*MHC*) class II genes (haplotypes DR3-DQ2 and DR4-DQ8), cytotoxic T-lymphocyte-associated protein 4 (*CTLA4*), protein tyrosine-phosphatase non-receptor type 22 (*PTPN22*), MHC class II transactivator (*CIITA*) [19, 24, 26], and the autoimmune regulator (*AIRE*) gene, which causes an autoimmune polyglandular syndrome that includes AD [24]. Although canine pedigree studies have indicated a recessive autosomal mode of inheritance for AD in the Standard Poodle and PWD [1, 15], a recent genome-wide association study (GWAS) on Standard Poodles failed to demonstrate significant associations with AD, suggesting a more complex mode of inheritance [27]. Candidate gene approaches have implicated many of the same genes identified in human AD with increased susceptibility to canine AD: *CTLA4* in the PWD, Cocker Spaniel and Springer Spaniel [10, 14, 28]; *PTPN22* in the Cocker Spaniel [10]; and the canine MHC (or dog leukocyte antigen, DLA) class II genes in the NSDTR, Bearded Collie and Standard Poodle [29–31]. Particular single nucleotide polymorphisms (SNPs) in *CTLA4* and collagen type IV alpha 4 chain (*COL4A4*) have been associated with AD in the Springer Spaniel, whereas SNPs in oxysterol binding protein like 9 (*OSBPL9*) and *PTPN22* were implicated in the Cocker Spaniel, and SNPs in syntaxin-binding protein 5 (*STXBP5*) in the Labrador Retriever. Two different genes (the vitamin D binding protein group-specific component (*GC*) and the SP110 nuclear body protein (*SP110*)) were investigated for association with AD in Bearded Collies, though no statistical significance was seen after correcting for multiple testing [32].

Taken together, these studies indicate the existence of genetic commonalities and breed-specificity in canine AD, underscoring the need for additional research to elucidate the genetic basis of this disease in dogs. A 2019 health survey conducted by the Bearded Collie Foundation for Health showed a relatively high prevalence of autoimmune conditions in the breed (11.1% based on 3072 Bearded Collies included in the survey). Of these, 3.1% had been diagnosed with primary hypoadrenocorticism [33]. Therefore, the present study aimed to identify genetic loci associated with AD in Bearded Collies by interrogating the genome. Given the likely polygenic nature of canine AD, we hypothesized that more than one genomic region would be associated with disease development and that those associated regions would harbor genes related to immune function and regulation.

## Results

### Genome-wide association study

**Fig. 1:**
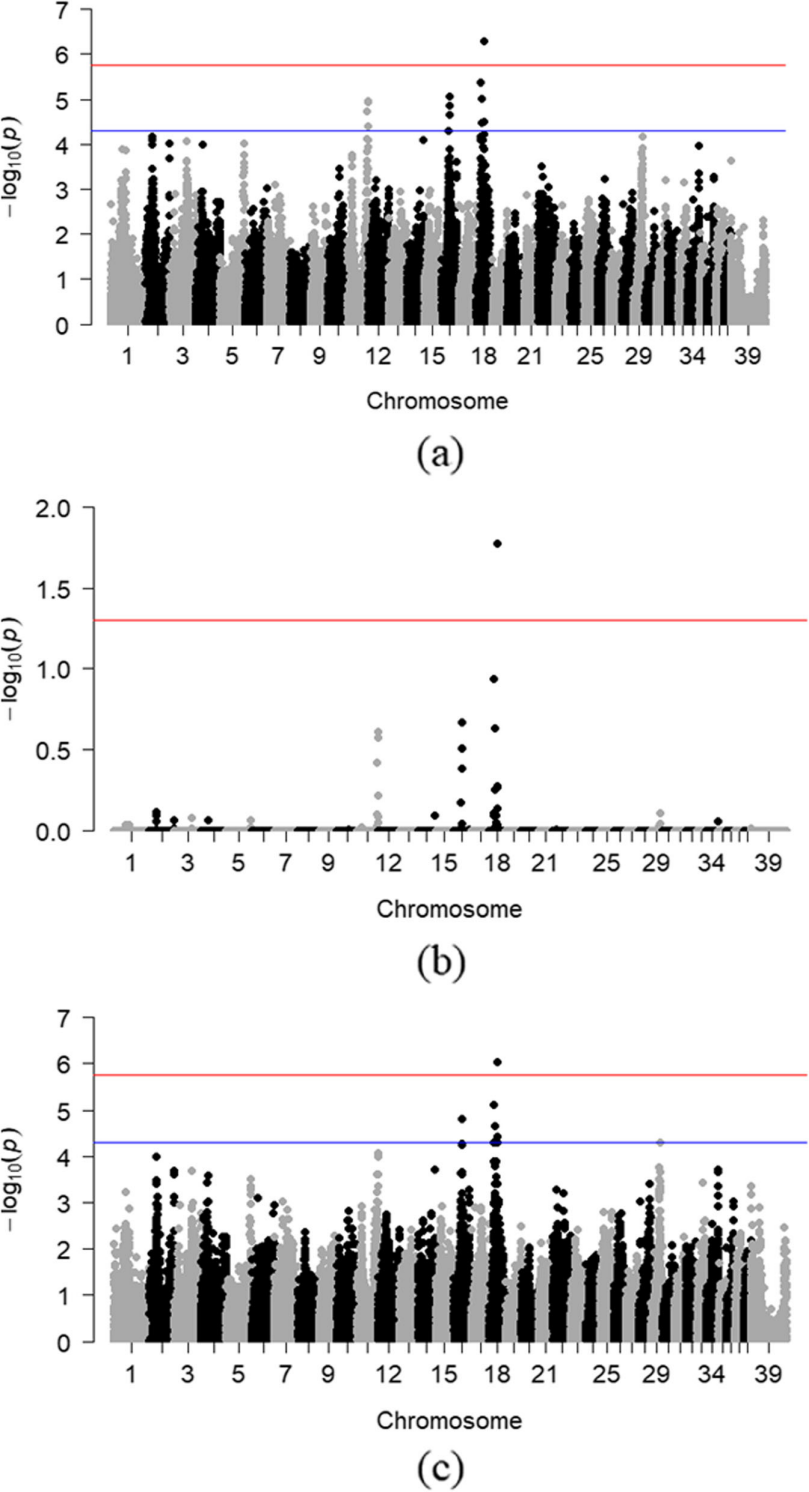
Manhattan Plots

PLINK and GEMMA analyses on unrelated dogs identified a peak on canine chromosome (CFA) 18 (Fig. 1a and b), with a single SNP (BICF2P1230367 at 28,207,384 bp) reaching genome-wide significance.

Chi-square based allelic association (A) and association testing after 100,000 max(T) permutations (B) in PLINK for 103 unrelated Bearded Collies (41 cases, 62 healthy controls; λ_GC_ = 1.2280). (C) Association testing using GEMMA’s univariate linear mixed model approach to account for population substructure of the same dataset (λ_GC_ = 1.0328). The blue and red lines indicate suggestive (*p* < 0.00005; −log10[*p*-value] ≥ 4.3) and Bonferroni-adjusted genome-wide significance threshold (−log10[*p*-value] ≥ 5.75), respectively.

To complement the findings in the GWA analyses, 37 additional minimally related dogs (3 male and 11 female AD cases, 11 male and 12 female controls) were genotyped for the top CFA18 SNP and the odds ratios (OR) calculated (Table 1; Additional Table 1); the total number of males and females analyzed across the cases and controls did not significantly differ (*p* > 0.05). Two genotypes were associated with increased risk for AD on CFA18; dogs homozygous for the alternate genotype (AA) or those with a heterozygous (AG) genotype were at higher risk for disease than those carrying a homozygous CanFam3.1 reference (GG) genotype (OR = 5.8, 95% CI = 2.62–12.92, *p* = 0.00001, and OR = 4.7, 95% CI = 1.53–14.70, *p* = 0.00742, respectively).

**Table 1:** Odds Ratio for the CFA18 SNP Significantly Associated with Addison’s Disease

CFA18 SNP (BICF2P1230367–28,207,384; CanFam3.1 reference allele G)^#^				
Genotype	Controls (*N* = 85)	AD cases (*N* = 55)	OR (95% CI)^*^	*p*-value
A A	7	9	2.2 (0.76–6.25)	0.17613
G A	19	30	**4.2 (2.00–8.70)**	**0.00013**
G G	59	16	**0.2 (0.09–0.38)**	**4.72 × 10**^**−6**^
A A	7	9		
vs			**4.7 (1.53–14.70)**	**0.00742**
G G	59	16		
G A	19	30		
vs			**5.8 (2.62–12.92)**	**0.00001**
G G	59	16		

Odds ratio (OR) for the CFA18 single nucleotide polymorphism (SNP) associated with Addison’s disease (AD) in 140 Bearded Collies (55 AD cases, 85 healthy controls). Bolded values were statistically significant at *p* < 0.05.

*CI Confidence interval, ^#^SNP location is based on the CanFam3.1 reference genome

Recognizing the complex nature of AD, we anticipated smaller effect loci contributing to AD expression, though perhaps not reaching genome-wide significance. A suggestive threshold was therefore set at *p* ≤ 0.00005 [34, 35]. Peaks of suggestive association were noted on chromosomes 11, 16 and 29. The GEMMA analyses yielded similar observations after accounting for the genetic structure of the sample (Fig. 1c). Specifically, the same top SNPs were observed in both PLINK and GEMMA analyses, although the SNP on CFA11 did not quite reach the suggestive threshold in the latter. Odds ratios were also calculated for these suggestive SNPs in all 140 dogs, which included the additional 37 dogs mentioned above (Table 2).

**Table 2:** Odds Ratio for the Top Suggestive SNPs on CFAs 11, 16, and 29

Genotype	Controls (*N* = 85)	AD cases (*N* = 55)	OR (95% CI)^*^	*p*-value
CFA11 SNP (BICF2G630307993–71,446,591; CanFam3.1 reference allele A)^#^				
G G	1	11	**21.0 (2.63–167.99)**	**0.00015**
G A	29	23	1.4 (0.69–2.79)	0.37576
A A	55	21	**0.3 (0.17–0.68)**	**0.00303**
G G	1	11		
vs A A	55	21	**28.8 (3.50–237.15)**	**0.00004**
G A	29	23		
vs			2.1 (0.99–4.37)	0.06010
A A	55	21		
CFA16 SNP (BICF2G630109748–27,126,780; CanFam3.1 reference allele C)				
G G	40	43	**4.0 (1.87–8.70)**	**0.00038**
C G	35	12	**0.4 (0.18–0.86)**	**0.02728**
C C	10	0	**NA**^**§**^	**0.01403**
G G	40	43		
vs			**NA**	**0.00151**
C C	10	0		
C G	35	12		
vs			NA	0.09974
C C	10	0		
CFA29 SNP (BICF2P977298–30,615,809; CanFam3.1 reference allele A)				
C C	16	27	**4.2 (1.95–8.88)**	**0.00019**
C A	47	20	**0.5 (0.23–0.93)**	**0.03760**
A A	22	8	0.5 (0.20–1.19)	0.14087
C C	16	27		
vs			**4.6 (1.68–12.85)**	**0.00397**
A A	22	8		
C A	47	20		
vs			1.2 (0.45–3.07)	0.81259
A A	22	8		

Odds ratio (OR) for the top suggestive single nucleotide polymorphisms (SNPs) on chromosomes 11, 16, and 29 in 140 Bearded Collies (55 Addison’s disease (AD) cases, 85 healthy controls). Bolded values were statistically significant at *p* < 0.05.

^§^NA not enough data points collected for OR calculation,^*^CI Confidence interval, ^#^SNP locations are based on the CanFam3.1 reference genome

A homozygous alternate genotype was associated with increased risk for AD at both CFA16 (i.e. GG) and CFA29 (i.e. CC) SNPs, both contributing similar risk for disease (OR = 4.0, 95% CI = 1.87–8.70, *p* = 0.00038; and OR = 4.2, 95% CI = 1.95–8.88, *p* = 0.00019, respectively). The highest OR obtained was associated with the homozygous alternate genotype on CFA11 (GG; OR = 21.0, 95% CI = 2.63–167.99, *p* = 0.00015). However, that risk genotype was observed in a single control dog and was only present in 20% of cases. Interestingly, all dogs carrying a risk genotype on CFA11 (GG) also carried the risk genotype on CFA16 (GG).

**Fig. 2:**
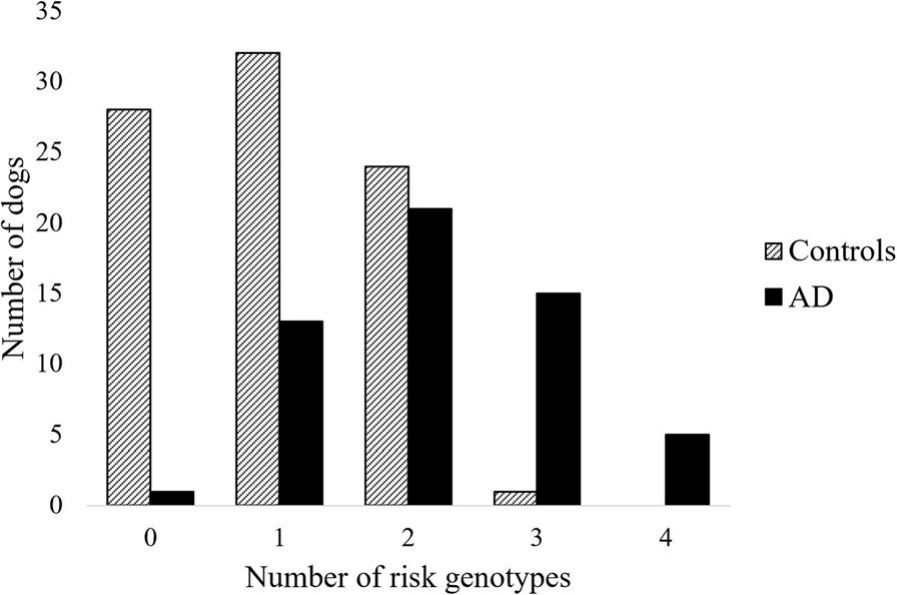
Number of Risk Genotypes in Case and Control Bearded Collies. Number of SNPs containing risk genotypes for Addison’s disease (AD) in 140 Bearded Collies (55 AD cases, 85 controls)

When the significant SNP on CFA18 and the suggestive SNPs on chromosomes 11, 16 and 29 were considered, as the number of risk genotypes increased, so did the risk for AD. That is, within these 140 dogs, dogs carrying risk genotypes at any one or more of these four evaluated SNPs were at greater risk for AD than dogs carrying only non-risk genotypes at all four SNPs (Fig. 2). Specifically, dogs carrying one or two risk genotypes were at greater risk for AD than dogs having none (OR = 17.0, 95% CI = 2.21–130.70, *p* = 0.00028), and dogs carrying risk genotypes at three or four of the SNPs were at greater risk for AD than those carrying one or two risk genotypes (OR = 32.9, 95% CI = 4.23–256.68, *p* = 9.03 × 10^− 7^).

To further investigate the observation that risk for AD increased with the number of risk genotypes a dog carried, logistic regression analysis was used to assess the genotypic contribution of each of the four SNPs of interest (the genome-wide significant CFA18 SNP and the suggestive SNPs on chromosomes 11, 16 and 29) on the probability of AD. Specifically, the analysis addressed the question of whether a multi-SNP model was better than the CFA18 single-SNP model in predicting the probability that a Bearded Collie would be affected by AD. Separate 2-SNP, 3-SNP and 4-SNP models were developed using the genotypes of the 55 Addisonian and 85 healthy control Bearded Collies. Lower leave-one-out information criterion (looic) values were noted for the multi-SNP models compared to the single-SNP ones. The lowest looic value was associated with the 4-SNP model, indicating that this model presented the best and most parsimonious fit for AD prediction in the study population (Additional Table 2). Based on the known genotypes and associated phenotypes, logistic regression calculated the probability of disease associated with every possible genotypic combination, even those that were not included in the data set (Additional Fig. 1), but only those associated with the highest risk genotypes are presented in the subsequent tables.

Taking the probability of disease associated with a CFA18 risk genotype as baseline, the addition of one other risk genotype at any of the three suggestive SNPs, in a 2-SNP interaction model, tended to increase the probability for AD, although a significant increase over the CFA18 SNP alone was only noted with the addition of a risk genotype on CFA11 (Table 3).

**Table 3:** Probability of Disease Based on Number of Risk Genotypes

Number of SNPs in model	CFA18	CFA11	CFA16	CFA29	Controls (N = 85)	AD (N = 55)	Probability of disease	95% CI*
Single SNP	A A	–	–	–	7	9	0.540	0.328–0.747
Single SNP	A G	–	–	–	19	30	0.613	0.479–0.744
2-SNP	A A	G G	–	–	1	1	0.877	0.637–0.988
2-SNP	A G	G G	**–**	**–**	0	**7**	**0.925**	**0.770–0.991**
2-SNP	A A	–	G G	–	4	8	0.641	0.407–0.834
2-SNP	A G	–	G G	–	11	22	0.697	0.552–0.824
2-SNP	A A	–	–	C C	1	5	0.757	0.524–0.915
2-SNP	A G	–	–	C C	2	14	0.822	0.677–0.924
3-SNP	A A	G G	G G	–	1	1	0.885	0.653–0.988
3-SNP	A G	G G	G G	–	0	7	**0.926**	**0.779–0.991**
3-SNP	A A	G G	–	C C	0	1	**0.950**	**0.803–0.996**
3-SNP	A G	G G	–	C C	0	4	**0.973**	**0.902–0.998**
3-SNP	A A	–	G G	C C	0	5	0.833	0.619–0.952
3-SNP	A G	–	G G	C C	0	11	**0.872**	**0.750–0.953**
4-SNP	A A	G G	G G	C C	0	1	**0.956**	**0.821–0.997**
4-SNP	A G	G G	G G	C C	0	4	**0.974**	**0.901–0.998**

Probability of disease associated with carrying one, two or three risk genotypes at the suggestive single nucleotide polymorphisms (SNPs) on chromosomes 11, 16 and/or 29 in addition to a CFA18 risk genotypes for Addison’s disease (AD) in 140 Bearded Collies (55 AD cases, 85 healthy controls). Comparisons provided are for genotypes associated with significant risk as detailed in Table 2. Bolded values indicate significant increase in the probability of disease associated with the additive effect of risk genotypes across 2 SNPs, 3 SNPs and all 4 SNPs of interest.

* *CI* Confidence interval

In contrast with the 2-SNP model, when considering a 3-SNP model, the addition of a risk genotype at any two suggestive SNPs also tended to increase the probability of AD in dogs carrying a risk genotype on CFA18, with significant increases noted for almost all possible 3-SNP risk genotype combinations. For the 4-SNP model, the addition of all four SNPs did not substantively increase the estimate of AD probability over the 3-SNP model likely reflecting that dogs that had a risk genotype on CFA11 also had the risk genotype on CFA16. Conversely, the probability of disease associated with carrying only non-risk genotypes at all four SNPs was extremely low for almost all possible 4-SNP combinations (Table 4).

**Table 4:** Probability of Disease for Dogs Carrying only Non-risk Genotypes

SNP Genotypes				Number of Dogs with Genotype Combination in Dataset		Probability of Disease	
CFA18	CFA11	CFA16	CFA29	Controls (N = 85)	AD cases (N = 55)	Probability	95% CI*
G G	A A	C C	A A	0	0	0.024	0.002–0.087 ^a^
G G	A A	C C	C A	3	0	0.021	0.002–0.079
G G	A A	C G	A A	3	0	0.067	0.016–0.170
G G	A A	C G	C A	10	0	0.056	0.014–0.131
G G	G A	C C	A A	0	0	0.061	0.006–0.209 ^a^
G G	G A	C C	C A	2	0	0.052	0.005–0.190
G G	G A	C G	A A	3	0	0.157	0.044–0.355
G G	G A	C G	C A	7	1	0.134	0.041–0.558

The table shows the probability of Addison's disease (AD) for dogs carrying only non-risk genotypes at all four single nucleotide polymorphisms (SNPs) of interest

*CI Confidence interval

^a^although none of the dogs in the dataset carried these genotypic combinations, logistic regression model allowed for the estimation of the probability of disease given the known genotypes and phenotypes.

### Haplotype analysis

**Fig. 3:**
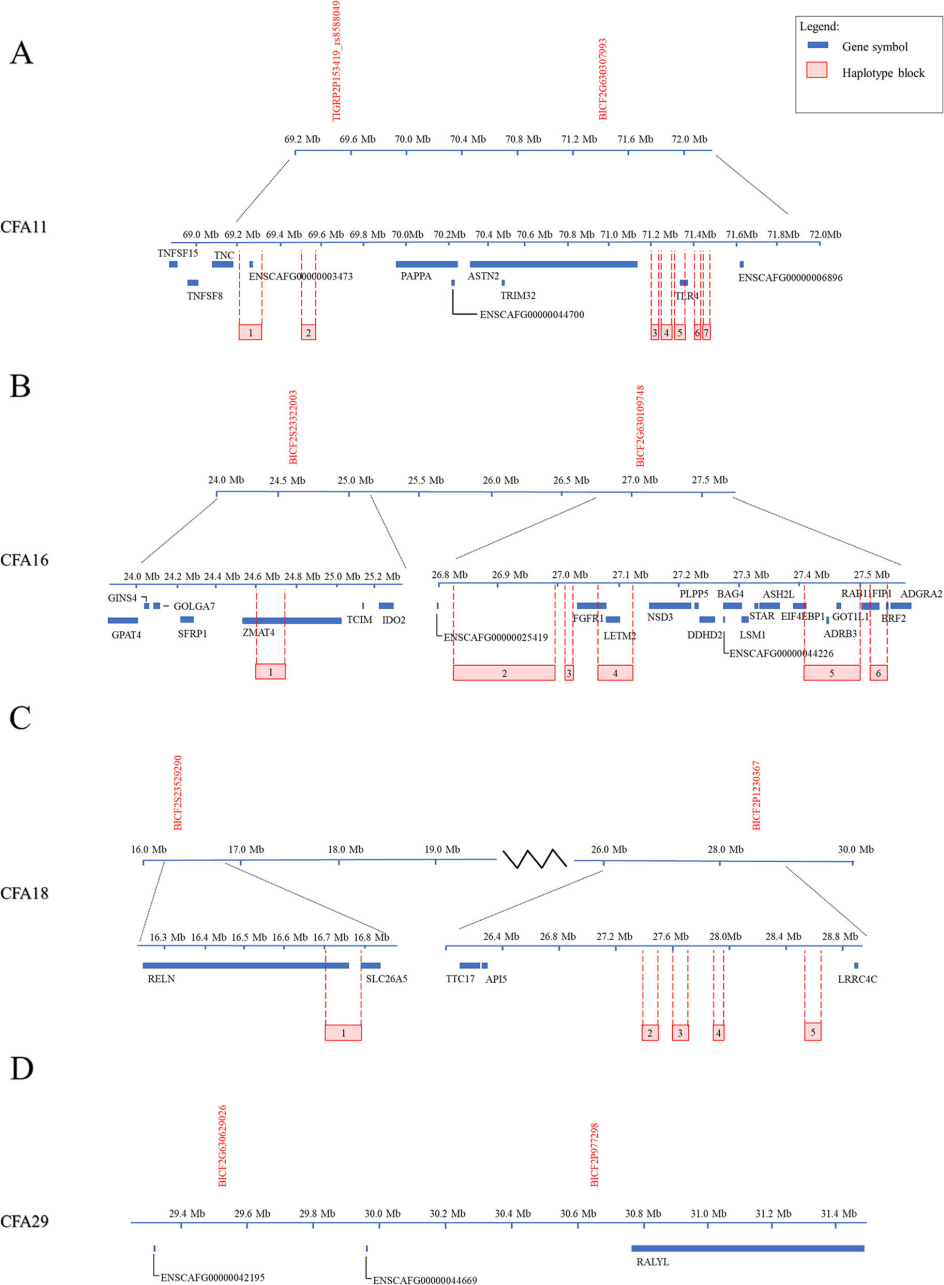
Location of Haplotype Blocks Associated with Addison’s Disease in Bearded Collies. Diagram showing the top GWAS SNPs and location of haplotype blocks on (A) CFA11, (B) CFA16, (C) CFA18 and (D) CFA29. No haplotype blocks were associated with Addison’s disease on CFA29. Top GWAS SNPs in each chromosome are listed in red

To refine the regions of association for candidate gene exploration, haplotype analysis was done for the four chromosomes. Eighteen haplotype blocks were significantly associated with AD (seven on CFA11, six on CFA16 and five on CFA18; permuted *p*-value< 0.05; Additional Table 3), and seven of them overlapped protein-coding genes. The location and distribution of the seven haplotype blocks on CFA11 indicate the existence of two associated regions separated by less than 2 Mb on CFA11 (Fig. 3a): one at the 69 Mb range and the other at 71 Mb. Moreover, one of the haplotype blocks on CFA11 overlapped with a protein-coding gene of utmost importance to the immune system: the toll-like receptor 4 (*TLR4*) gene.

Similar to CFA11, five haplotype blocks on CFA16 clustered around the 27 Mb range (Fig. 3b), coinciding with the location of the top GWAS SNP (located within haplotype block 4), whereas a sixth haplotype block was located approximately 2 Mb upstream of that region. Moreover, four of the haplotype blocks overlapped protein-coding genes: zinc finger matrin-type 4 (*ZMAT4*), fibroblast growth factor receptor 1 (*FGFR1*), leucine zipper and EF-hand containing transmembrane protein 2 (*LETM2*), eukaryotic translation initiation factor 4E binding protein 1 (*EIF4EBP1*), adrenoreceptor beta 3 (*ADRB3*), glutamic-oxaloacetic transaminase 1 like 1 (*GOT1L1*), RAB11 family interacting protein 1 (*RAB11FIP1*) and BRF2 RNA polymerase III transcription initiation factor subunit (*BRF2*).

Unlike with chromosomes 11 and 16, none of the haplotype blocks on CFA18 contained the top GWAS SNP (Fig. 3c), which is located about half-way between haplotype blocks 4 and 5. Four of the haplotype blocks were clustered around the 27 Mb range, spanning a total of 1.2 Mb in a region containing various RNA genes and few protein-coding genes. The fifth block was located almost 11 Mb upstream of that region and overlapped with two protein-coding genes: reelin (*RELN*) and solute carrier family 26 member 5 (*SLC26A5*). No haplotype blocks were associated with AD on CFA29 (Fig. 3d).

## Discussion

The present study reports the first genome-wide association analysis to identify a statistically significant region of association with canine AD. Three suggestive smaller effect loci were also identified and shown to contribute to increased risk for AD in Bearded Collies. Previous studies focused on candidate gene approaches yielded few associations, most of which appear to be breed specific [2, 9, 10, 28, 32, 36]. Similar to human AD [24], MHC (or DLA) class II genes have shown the most consistent associations with canine AD to date, even though different DLA class II haplotypes have been associated with increased risk for the disease in different breeds [9, 29–31]. However, DLA class II haplotypes fail to completely explain AD development in both dogs and humans, and only 37.7% of Bearded Collies with AD possess the breed’s identified DLA class II risk haplotype for the disease [29]. This may explain the lack of association with the DLA class II locus on CFA12 in the present study and underscores the importance of identifying additional loci contributing to AD risk.

Although sample size was a limitation of this study, a genome-wide significant region of association was identified on CFA18, revealing two risk genotypes for the top CFA18 SNP (AA and AG). Given the complex nature of canine AD, a single major locus associated with disease risk was not expected. Instead, complex traits are likely governed by multiple loci of smaller effects in addition to environmental factors, and SNPs representing these smaller effect genomic regions are not likely to reach genome-wide significance [35]. Peaks reaching the suggestive threshold for disease association were identified on chromosomes 11, 16, and 29, and a homozygous alternate genotype at the top SNP of each chromosome was associated with increased risk for AD.

Grouping of the dogs based on the number of risk genotypes they carried at all four SNPs indicated that, as the number of risk genotypes increased, so did the risk for AD. Logistic regression analysis further supported a contribution of additional risk genotypes at these smaller effect loci (on chromosomes 11, 16 and 29) to the probability of disease associated with a risk genotype on CFA18, with a 4-SNP model presenting the best fit for predicting the probability of AD in the study population although a 3-SNP model showed similar performance and captured a greater number of AD cases. Notably, despite presenting the best fit, only ~ 10% of AD dogs carried risk genotypes at all 4 SNPs. While one might anticipate a greater number of AD cases to be captured by such a comprehensive model, especially when the study cohort consisted of a single dog breed expected to be genetically similar, complex inherited diseases such as AD may not require the presence of every single risk locus for disease development.

It should also be mentioned that the small sample size meant that many possible genotypic combinations were not represented in the dataset. Nevertheless, logistic regression analysis still allows for prediction of disease risk for all plausible genotype combinations, recognizing that the predictions in these “empty cells” is not very precise and the confidence intervals surrounding the probability estimates will be large. Based upon our assessment of genotype effects at these four chromosomes, there were genotype combinations that could be considered high risk for AD and others showing strong evidence of being associated with dogs not afflicted with AD, as shown in Additional Fig. 1. However, the amount of risk conferred by each SNP, or combinations of SNPs, remains unclear.

The highest OR was associated with a homozygous alternate genotype for the top CFA11 SNP, and all 2-SNP, 3-SNP and 4-SNP genotypic combinations showing the highest probability of disease carried the CFA11 risk genotype. However, this genotype was only seen in 20% (11/55) of cases, which might suggest heterogeneity of our case group despite our strict inclusion criteria. Given that all dogs carrying the risk genotype on CFA11 also carried a risk genotype on CFA16, it is more likely that the very high risk for AD seen in dogs carrying the CFA11 risk genotype is due to an additive effect of the two risk genotypes. This fact may also have confounded the 3-SNP and 4-SNP models, and may be why no significant differences were seen between the probability of disease in dogs carrying three or four risk genotypes.

The genome-wide significant SNP identified in our study lands in a gene-poor region of CFA18. The closest protein-coding gene is *LRRC4C* (leucine rich repeat containing 4C, also known as netrin-G1 ligand, NGL-1), located approximately 600 kb downstream of the SNP and involved in thalamocortical axon guidance during embryonic development [37]. No other protein-coding genes were identified within 1 Mb upstream or downstream of the SNP. Based upon the literature, *LRRC4C* has no connection to immune function, inflammation, or adrenal development. However, long noncoding RNA (lncRNA) genes can be found approximately 500Kb upstream and downstream of the associated SNP. Long noncoding RNAs have limited or no protein-coding capacity, but can regulate various biological processes, including immune cell development and function. These molecules can also act as transcriptional regulators of neighboring genes, and recent studies have implicated lncRNAs in autoimmune diseases such as systemic lupus erythematosus (SLE) and rheumatoid arthritis (RA) [38]. Gene-poor regions of the genome have also been shown to harbor regulatory sequences that can bind transcription factors and regulate gene expression. In fact, more than half of the GWAS variants associated with human autoimmune diseases fall in enhancer elements, attributing much of the genetic risk for autoimmune conditions to the modulation of gene expression [39]. Whereas the mapping of regulatory elements to the canine genome is not yet available, research teams affiliated with The Dog Genome Annotation Project (DoGA) have been working to generate such functional annotation to facilitate the study of canine diseases.

Haplotype analysis on CFA18 indicated a second region of association approximately 12 Mb upstream of the genome-wide significant SNP. The haplotype block identified in this region overlaps two genes *RELN* and *SLC26A5*. However, based on the literature, neither appear to be potential candidate genes for canine AD. *SLC26A5* encodes an anion transporter molecule responsible for electromotility in mammalian auditory sensory hair cells [40], and mutations in the human gene have been associated with hearing loss (autosomal recessive deafness; OMIM 604943). *RELN* plays a critical role in brain formation, and mice with Reln deficiency (i.e. reeler mice) present with malformations of the cerebral cortex [41]. Though immune abnormalities have been reported in reeler mice, they were not compatible with autoimmune diseases and characterized by reduced cytokine production, T cell proliferation and B cell responses [42].

The top CFA11 SNP was located within a lncRNA gene adjacent to *TLR4*. As mentioned above, lncRNAs may be involved in autoimmunity through the regulation of neighboring genes [38]. Moreover, five haplotype blocks were identified in that region, one of which overlapped with *TLR4*. TLR4 is a membrane-bound pattern recognition receptor expressed in macrophages, dendritic cells and T lymphocytes, and binding of this receptor results in the activation of signaling pathways that lead to the production of pro-inflammatory cytokines, chemokines and costimulatory molecules that activate the adaptive immune response [43]. Dysregulation of TLR4 signaling may result in overt activation of proinflammatory signals, thus increasing the risk for autoreactive lymphocyte activation. Autoimmune diseases that have been associated with TLR4 include RA, SLE and type 1 diabetes [44].

Two haplotype blocks associated with AD were also identified approximately 1.5 Mb upstream of the top CFA11 GWAS SNP. Two immune genes can be found within 50Kb upstream of the haplotype blocks: TNF superfamily member 15 (*TNFSF15*) and TNF superfamily member 8 (*TNFSF8*). TNFSF15 is a member of the tumor necrosis factor family of ligands that can influence the proliferation, activation and differentiation of immune cells, including T cells. TNFSF15 expression can be induced in monocytes, macrophages and dendritic cells through TLR signaling, for instance, and in T lymphocytes in response to T-cell receptor (TCR) binding under inflammatory conditions [45]. Polymorphisms in this gene have been associated with several autoimmune diseases, such as RA, inflammatory bowel disease, Crohn’s disease [46], SLE, and Grave’s disease – a polygenic organ-specific autoimmune thyroid condition [47]. Similarly, TNFSF8 (also known as CD30 ligand) also plays a role in modulating T cell responses, where binding of these ligands on T lymphocytes results in augmented cell proliferation and cytokine production [48]. Despite its recognized potential role in autoimmunity, to the best of our knowledge, no associations between polymorphisms in *TNFSF8* have been reported for any specific autoimmune disorder.

The top SNP on CFA16 was intergenic and located in a gene-rich region of the chromosome. Similar to chromosomes 11 and 18, haplotype blocks associated with AD suggested two regions of association approximately 1 Mb apart on CFA16. Four of the six haplotype blocks overlapped with protein-coding genes: *ZMAT4*, *FGFR1*, *LETM2*, *EIF4EBP1*, *GOT1L1*, *RAB11FIP1*, *BRF2* and beta-3 adrenergic receptor (*ADRB3*). However, literature review showed no evidence of their direct connection to immune function or adrenal development, making them unlikely candidates for susceptibility to canine AD. While *EIF4EBP1* and *FGFR1* are involved in the PI3K-Akt signaling pathway, which does play a role in regulating immune cell effector functions through various mechanisms [49], a more likely candidate gene for AD susceptibility would be indoleamine 2,3-dioxygenase 2 (*IDO2),* one of two neighboring genes involved in tryptophan catabolism and modulation of immune responses. The better characterized gene, *IDO1*, is known to modulate the immune system by altering regulatory T cell populations. Although *IDO2* is less efficient at catabolizing tryptophan, it is expressed in antigen presenting cells and has been recently implicated in driving B-cell mediated and T cell-dependent autoimmune responses [50, 51].

It should also be noted that a gene involved in steroidogenesis can be found 200Kb downstream of the top CFA16 SNP: the steroidogenic acute regulatory protein (*STAR*) gene. This gene encodes a mitochondrial transporter protein that transfers cholesterol from the outer to the inner mitochondrial membrane where steroidogenesis is initiated. Various inactivating mutations in *STAR* result in lipoid congenital adrenal hyperplasia, a severe (and usually fatal) condition that manifests within the first weeks to months of life. Severe *STAR* deficiency also causes failed testosterone production in utero resulting in males being born with female genitalia [52]. While inactivating mutations in *STAR* are extremely unlikely to be involved in canine AD, normal steroidogenesis in the adrenal glands is thought to suppress antigen presentation within the adrenal cortex [19]. It is, therefore, possible that mutations in *STAR* causing even a mild reduction in steroidogenesis could promote a breakdown of tolerance to adrenocortical antigens, thus facilitating the onset of autoimmunity.

No haplotype blocks associated with AD were identified on CFA29. The two top SNPs on that chromosome marked a 1 Mb region of the chromosome that contained three protein-coding genes, two of which were uncharacterized. No potential candidate genes for canine AD were identified in the associated region of this chromosome.

Susceptibility to complex human autoimmune diseases, including AD, is commonly associated with an accumulation of small effect variants in multiple immune-related genes that are usually insufficient to cause disease on their own [53]. Moreover, complex traits such as AD are influenced by environmental factors that may act as triggers for disease expression in some but not all carriers of risk genotypes. Though based upon a relatively small sample size, our findings are consistent with these observations, and suggest a combination of risk genotypes in different genomic loci contributing to AD development in Bearded Collies. While a larger sample size may help further elucidate the contributions of each associated region, it is unlikely that we would be able to expand the sample to any great degree given the relatively small actual and effective population size of Bearded Collies [54]. Incomplete penetrance and potential genetic heterogeneity of canine AD may also limit our ability to determine the amount of risk each region confers. Low prevalence, complex nature, incomplete penetrance and potential heterogeneity makes it challenging to investigate the genetic basis of human AD [19], and similar challenges exist with canine AD, even within predisposed dog breeds. Nonetheless, our study offers progress in the identification of susceptibility loci for canine AD in Bearded Collies.

## Conclusions

Despite the small sample size, the present study identified a genome-wide significant region of association with canine AD on CFA18, in addition to suggestive regions of association on chromosomes 11, 16 and 29. Logistic regression supported an additive contribution of risk genotypes at each of the associated loci to the probability of developing AD in Bearded Collies. Associated loci overlap with potential candidate genes for AD susceptibility that have roles regulating adrenal steroidogenesis, immune responses and/or inflammation. These findings offer progress in the identification of susceptibility loci for canine AD Bearded Collies, though further studies are needed to confirm association of the identified candidate genes and determine actual causative mutations involved with AD susceptibility in this breed.

## Methods

### Samples

Blood or buccal swab samples from privately owned Addisonian and healthy Bearded Collies were submitted to our laboratory by the dog’s owner or veterinarian. Addisonian dogs (AD cases) included in this study were selected based on a veterinary diagnosis of primary hypoadrenocorticism that included an ACTH stimulation test (serum cortisol levels < 55 nmol/L before and after ACTH administration) and evidence of electrolyte imbalance (sodium to potassium ratio < 27:1) and had no other concurrent illnesses (diagnosed or suspected). The AD cases had an average age of AD diagnosis of 50 months (range 5 to 120 months). Dogs included as healthy controls were at least 10 years old (average 12.5 years), with no history of AD or other autoimmune diseases (diagnosed or suspected). Samples were subjected to DNA extraction as previously described [55], and DNA aliquots quantified using a Nanodrop® spectrophotometer and stored at − 20 °C until further analysis.

### Genome-wide association study

One hundred and forty-three DNA samples, consisting of 59 AD cases (22 males, 37 females) and 84 controls (34 males, 50 females), were genotyped at GeneSeek (Lincoln, NE) using the Illumina CanineHD BeadChip (San Diego, CA) containing 173,662 SNPs. Reference alleles and SNP locations were based on the CanFam3.1 reference genome; alleles differing from the reference allele at any given location were referred to as alternate alleles. Individuals and SNPs with less than 95% call rates as well as SNPs with minor allele frequency less than 1% were removed from the dataset using PLINK version 1.9 [56]. Autosomal SNPs with a minor allele frequency greater than 1% were then used to generate a genetic relationship matrix for the remaining dogs using GCTA [57]; closely related individuals were excluded from GWAS analysis based on a relatedness cutoff of 0.3. Of the 143 dogs that underwent GWA analyses, 36 (17 AD cases, 19 controls) were removed from the analysis based on a relatedness cutoff of 0.3, and four dogs (one AD case and three controls) were excluded from analysis due to poor genotyping. Association analyses using the 98,540 SNPs remaining after quality control was done on the 103 unrelated dogs (41 AD cases and 62 controls) using PLINK version 1.9 [56] following exclusion of SNPs that deviated from Hardy-Weinberg equilibrium among controls (*p* > 0.0001) and/or with a minor allele frequency less than 5%. The univariate linear mixed model approach implemented by GEMMA [58] was also used to account for any remaining population structure. As previously noted [34], most of the SNPs used in a canine GWAS are in linkage disequilibrium with multiple other SNPs, thus the number of SNPs used for analysis does not accurately represent the number of independent tests. To avoid overcorrecting for multiple testing, genome-wide significance was established using a Bonferroni-corrected alpha value of 0.05 divided by the effective number of independent SNPs as determined by GEC (Genetic type 1 error calculator) [59]. Based on published studies addressing the genetics of complex traits in dogs, a threshold for suggestive association was set at *p* ≤ 0.00005 [34, 35].

### Haplotype analysis

To narrow down regions of interest for candidate gene exploration, haplotype analysis of the GWAS data was done using Haploview version 4.2 [60] and 25,000 permutations on the chromosomes containing SNPs with suggestive and/or genome-wide significant association with AD. Genes overlapping with haplotype blocks were explored for potential involvement in AD development.

### Genotyping of the four GWAS SNPs of interest on additional dogs

Polymerase chain reaction (PCR) was used to genotype significant and suggestive SNPs of interest (CFA11: BICF2G630307993, CFA16: BICF2G630109748, CFA18: BICF2P1230367 and CFA29: BICF2P977298) on additional dogs selected to be minimally related to those used for GWAS based on pedigree analysis, that is sharing no more than one grandparent. Primers flanking each of the SNPs were designed using Primer3 [61] (CFA11-F: TGCCACATTCCCTTCCTTCT, CFA11-R: AGCATGACAAGACAGGACGA; CFA16-F: TTGTTTTGTTGTGTTTCCCTCTT, CFA16-R: TATCACAGTAAGGGGCCATAGG; CFA18-F: TTGGTTGTGCGTAGTCCTCT, CFA18-R: TCATCGCTCTAGTCACTGGG; CFA29-F: GCTAAGTACGCCTTGCAACC, CFA29-R: CCTGTGCTTGGATGTGATTG). A standard 31 cycle PCR protocol was used with an annealing temperature of 60 °C for the CFA11, CFA16 and CFA18 primer sets and 62 °C for the CFA29 primers. A 25 μL reaction and Promega GoTaq® Flexi DNA Polymerase (Promega, WI, USA) were used. Amplicon size was confirmed by running 6 μL of the PCR product on a 1% agarose gel. Exosap-IT™ express (Thermo Fisher Scientific, Waltham, MA, USA) was used to purify the PCR products according to the manufacturer’s recommendations, and these were then sequenced by capillary electrophoresis on an ABI 3730 DNA analyzer (Applied Biosystems, Foster City, CA, USA). Sequences were read using FinchTV v.1.4 (Geospiza, USA) and genotypes obtained by aligning the sequences against the CanFam3.1 reference sequence using CLC viewer v.7.8.1 (QIAGEN Bioinformatics, Denmark).

### Statistical analysis

Odds ratio (OR) and two-tailed Fisher’s exact *p*-values were calculated using a 2 × 2 contingency table in VassarStats (http://vassarstats.net/odds2x2.html); OR calculations were based on the number of AD cases and healthy controls carrying a particular genotype compared to the number of cases and controls not carrying that genotype. Additionally, OR calculations included genotypic pairwise comparisons (i.e. homozygous alternate versus heterozygous; homozygous alternate versus homozygous reference; and heterozygous versus homozygous reference) to identify which genotypes conferred risk for AD. Statistical significance was set at *p* < 0.05.

Logistic regression was used to model the risk of disease as a function of the observed genotypes at each of the four SNPs of interest (CFA11: BICF2G630307993, CFA16: BICF2G630109748, CFA18: BICF2P1230367 and CFA29: BICF2P977298) across the unrelated dogs. Disease probability was defined by methods previously described [62]. Briefly, the logit of disease probability was modeled as a function of the SNP genotypes representing 3 classes (e.g., AA, AG and GG with 2 degrees of freedom in the analysis). Genotypic effects and predictions of disease risk were determined using the Bayesian statistical package Stan [63] executed in R [64]. A series of models were built using various combinations of the potentially important SNPs and the model with the best predictive accuracy for disease was identified using the leave-one-out cross-validation process [65], which calculates a statistic referred to as the leave-one-out information criterion (looic). Similar to that observed with the Akaike Information Criterion (AIC) for frequentist methods, the model with the smallest looic value is considered the best fit for predicting disease status. Looic calculation was performed for each of the models using the R package loo [66]. The receiver operating characteristic curve, fitted with the R package pROC [67], was then used to assess the value of the models in disease prediction and define the optimal discrimination threshold for assigning a healthy or AD status to dogs based on each individual’s known SNP genotypes.

## Supplementary Information

**Additional file 1 **: **Figure 1**. Logistic regression analysis for the four SNPs of interest. Probability of AD associated with the 81 plausible 4-SNP genotypic combinations based on known genotypes and phenotypes of 140 Bearded Collies (55 AD cases, 85 controls). Logistic regression calculates probabilities for genotypic combinations that are not included in the data set, so one can still predict the disease risk for all 81 plausible genotypes recognizing that the predictions in these “empty cells” is not very precise and the confidence intervals surrounding the probability estimates will be large. Based upon the observed data, probability estimates in excess of 0.42 were associated with AD risk whereas genetic combinations that had a probability below 0.42 were associated with reduced AD risk. Thus, in this figure, 0.42 was set as the baseline zero value. Probability estimates in excess of that figure are depicted above the line and those lower are depicted as below the line. For example, the highest probability calculated was 0.974, which would be represented as 0.55 above the threshold value of 0.42.

**Additional file 2 **: **Table 1.** Genotypes of the Bearded Collies analyzed at the four SNPs of interest. Genotypes of the 140 Bearded Collies (55 AD cases, 85 healthy controls) analyzed at each of the four SNPs of interest: BICF2G630307993 on canine chromosome (CFA) 11, BICF2G630109748 on CFA16, BICF2P1230367 on CFA18 and BICF2P977298 on CFA29.

**Additional file 3 **: **Table 2.** Model assessment. Values for the leave-one-out information criterion (looic) and standard error associated with each of the predictive SNP models tested. The model with the smallest looic value is considered the best, most parsimonious, fit.

**Additional file 4 **: **Table 3**. Haplotype blocks associated with Addison’s disease in Bearded Collies. Haplotype blocks associated with Addison’s disease on canine chromosomes 11, 16 and 18 in 103 unrelated Bearded Collies (41 cases, 62 controls). Locations represent the position of the first and last single nucleotide polymorphism (SNP) in each block and are based on the CanFam3.1 reference genome.

## Data Availability

All data generated or analyzed during this study are included in this published article and its supplementary information files.

## Abbreviations

AD: Addison’s disease
CFA: Canine chromosome;
GWAS: Genome wide association study
OMIA: Online Mendelian inheritance in animals
PWD: Portuguese Water Dog
NSDTR: Nova Scotia Duck Tolling Retriever
MHC: Major histocompatibility complex
CTLA4: Cytotoxic T-lymphocyte-associated protein 4
PTPN22: Protein tyrosine-phosphatase non-receptor type 22
CIITA: MHC class II transactivator
AIRE: Autoimmune regulator
SNP: Single nucleotide polymorphism
OSBPL9: Oxysterol binding protein like 9 gene
DLA: Dog leukocyte antigen
COL4A4: Collagen type IV alpha 4 chain
STXBP5: Syntaxin-binding protein 5
GC: Vitamin D binding protein group-specific component
SP110: SP110 nuclear body protein
OR: Odds ratio
CI: Confidence interval
LOOIC: Leave-one-out criterion
TLR4: The toll-like receptor 4
ZMAT4: Zinc finger matrin-type 4
FGFR1: Fibroblast growth factor receptor 1
LETM2: Leucine zipper and EF-hand containing transmembrane protein 2
EIF4EBP1: Eukaryotic translation initiation factor 4E binding protein 1
ADRB3: Adrenoreceptor beta 3
GOT1L1: Glutamic-oxaloacetic transaminase 1 like 1
RAB11FIP1: RAB11 family interacting protein 1
BRF2: BRF2 RNA polymerase III transcription initiation factor subunit
RNA: Ribonucleic acid
RELN: Reelin
SLC26A5: Solute carrier family 26 member 5
LRRC4C: Leucine rich repeat containing 4C
NGL-1: Netrin-G1 ligand
SLE: Systemic lupus erythematosus
RA: Rheumatoid arthritis
DoGA: The Dog Genome Annotation Project
OMIM: Online Mendelian inheritance in man
lncRNA: Long noncoding ribonucleic acid
TNFS15: TNF superfamily member 15
TNFSF8: TNF superfamily member 8
TCR: T-cell receptor
ADRB3: Beta-3 adrenergic receptor
IDO2: Indoleamine 2,3-dioxygenase 2
IDO1: Indoleamine 2,3-dioxygenase 1
STAR: Steroidogenic acute regulatory protein
ACTH: Adrenocorticotropic hormone
DNA: Deoxyribonucleic acid
PCR: Polymerase chain reaction
AIC: Akaike information criterion
