# Correction to: Genetic characterization of Addison’s disease in Bearded Collies

**DOI:** 10.1186/s12864-020-07328-w

**Published:** 2020-12-30

**Authors:** Liza C. Gershony, Janelle M. Belanger, Marjo K. Hytönen, Hannes Lohi, Thomas R. Famula, Anita M. Oberbauer

**Affiliations:** 1 Department of Animal Science, University of California-Davis, Davis, CA 95616 USA; 2 Brazilian National Council for Scientific and Technological Development (CNPq) fellow, Brasilia, DF 71605 Brazil; 3 Department of Medical and Clinical Genetics, and Department of Veterinary Biosciences, University of Helsinki, 00014 Helsinki, Finland; Folkhälsan Research Center, Helsinki, 00290 Finland

**Correction to: BMC Genomics (2020) 21:833**

**https://doi.org/10.1186/s12864-020-07243-0**

Following publication of the original article [1], it was noted that due to a typesetting error the caption of Fig. 1 was mistakenly captured within the main body of the article.

**Fig. 1:**
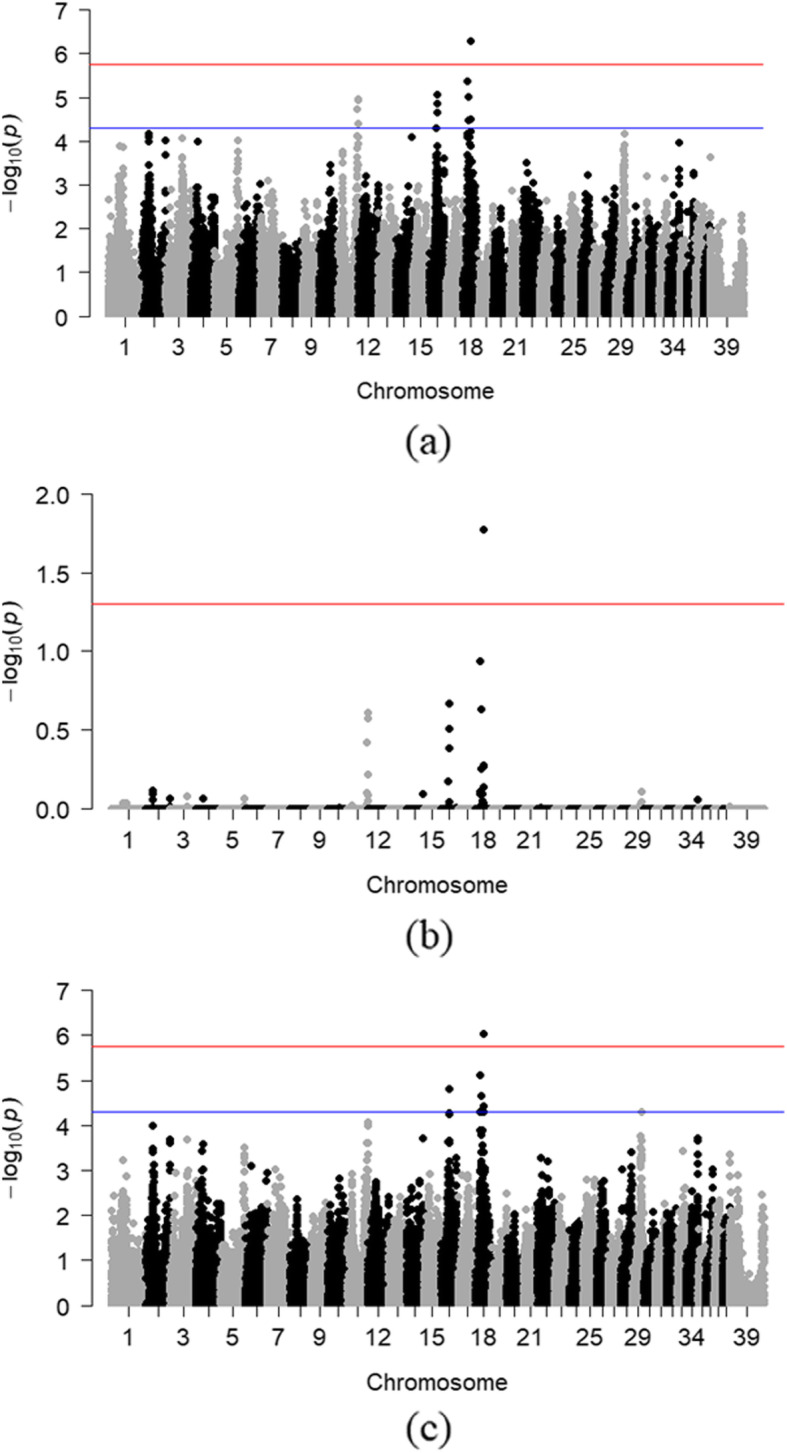
Manhattan Plots. Chi-square based allelic association (**a**) and association testing after 100,000 max(T) permutations (**b**) in PLINK for 103 unrelated Bearded Collies (41 cases, 62 healthy controls; λGC = 1.2280). **c** Association testing using GEMMA’s univariate linear mixed model approach to account for population substructure of the same dataset (λGC = 1.0328). The blue and red lines indicate suggestive (*p* < 0.00005; −log10[*p*-value] ≥ 4.3) and Bonferroni-adjusted genome-wide significance threshold (−log10[p-value] ≥ 5.75), respectively

Furthermore, the layout of the online versions of Tables 1 and 2 have been updated to improve the presentation of the genotype comparisons.

The correct Fig. 1 with the caption has been included in this Correction article, and the original article has been updated.
