# Supplementary figures and images for: Genetic characterization of Addison’s disease in Bearded Collies
Source: BMC Genomics. 2020 Nov 26;21:833. doi: 10.1186/s12864-020-07243-0 (PMC7690126; doi:10.1186/s12864-020-07243-0)

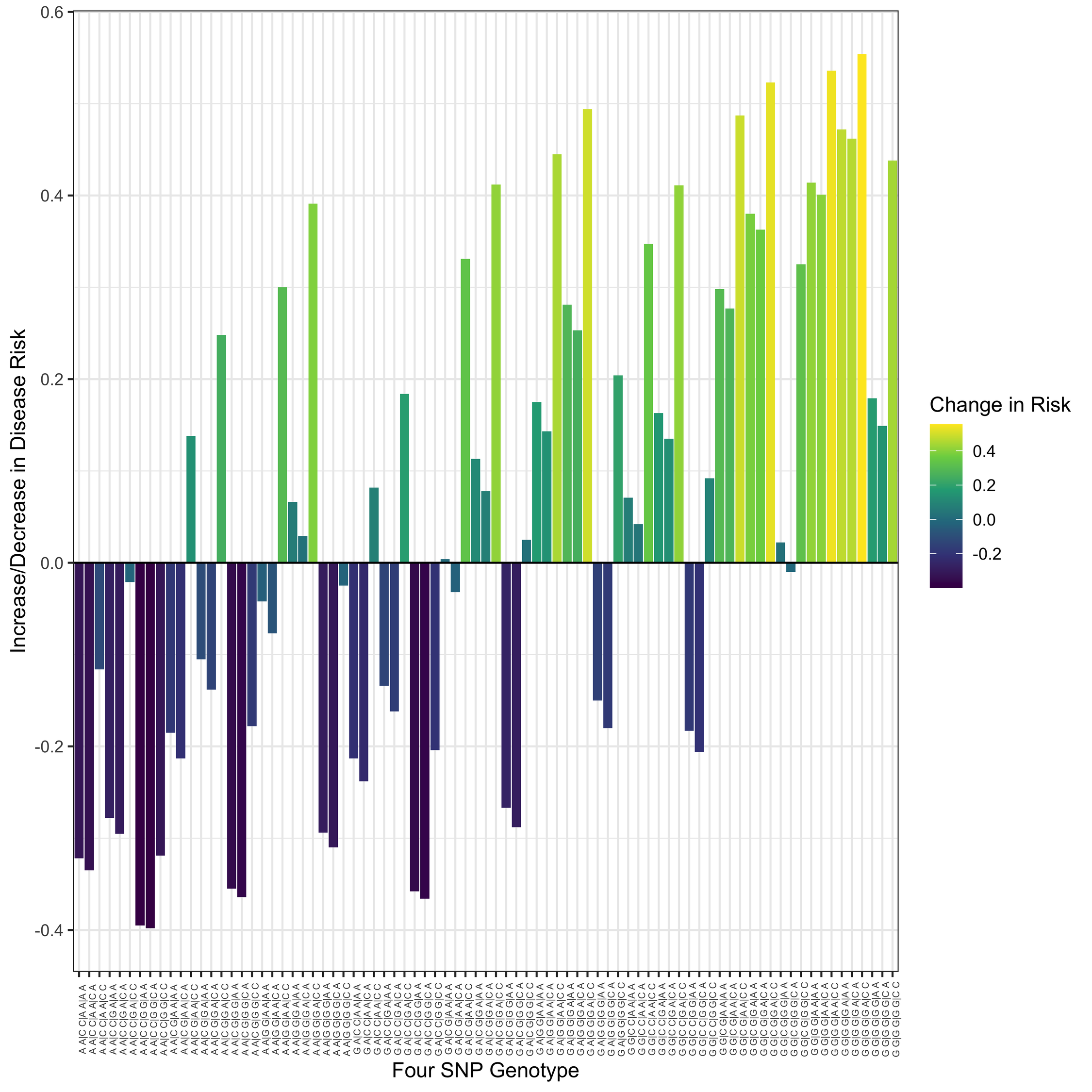

Supplement: Supplementary file 1 — Additional file 1 : Figure 1. Logistic regression analysis for the four SNPs of interest. Probability of AD associated with the 81 plausible 4-SNP genotypic combinations based on known genotypes and phenotypes of 140 Bearded Collies (55 AD cases, 85 controls). Logistic regression calculates probabilities for genotypic combinations that are not included in the data set, so one can still predict the disease risk for all 81 plausible genotypes recognizing that the predictions in these “empty cells” is not very precise and the confidence intervals surrounding the probability estimates will be large. Based upon the observed data, probability estimates in excess of 0.42 were associated with AD risk whereas genetic combinations that had a probability below 0.42 were associated with reduced AD risk. Thus, in this figure, 0.42 was set as the baseline zero value. Probability estimates in excess of that figure are depicted above the line and those lower are depicted as below the line. For example, the highest probability calculated was 0.974, which would be represented as 0.55 above the threshold value of 0.42. [file 12864_2020_7243_MOESM1_ESM.tif]
