# Supplementary material for: Genetic characterization of Addison’s disease in Bearded Collies
Source: BMC Genomics. 2020 Nov 26;21:833. doi: 10.1186/s12864-020-07243-0 (PMC7690126; doi:10.1186/s12864-020-07243-0)
Supplement: Supplementary file 2 — Additional file 2 : Table 1. Genotypes of the Bearded Collies analyzed at the four SNPs of interest. Genotypes of the 140 Bearded Collies (55 AD cases, 85 healthy controls) analyzed at each of the four SNPs of interest: BICF2G630307993 on canine chromosome (CFA) 11, BICF2G630109748 on CFA16, BICF2P1230367 on CFA18 and BICF2P977298 on CFA29. [file 12864_2020_7243_MOESM2_ESM.docx]

**Additional Table 1. Genotypes of the Bearded Collies analyzed at the four SNPs of interest.**

| **Dog ID** | **Sex** | **Status** | **11:BICF2G630307993** | **16:BICF2G630109748** | **18:BICF2P1230367** | **29:BICF2P977298** |
| --- | --- | --- | --- | --- | --- | --- |
| 1 | M | AD | G A | G G | A G | C C |
| 2 | M | CTRL | A A | G G | G G | C A |
| 3 | F | AD | G G | G G | A G | C A |
| 4 | F | AD | G G | G G | A G | C C |
| 5 | F | AD | A A | G G | A A | C C |
| 6 | F | CTRL | G A | C G | G G | C A |
| 7 | M | CTRL | A A | C G | G G | C A |
| 8 | F | AD | A A | G G | A G | C A |
| 9 | F | CTRL | A A | G G | A G | C A |
| 10 | F | AD | G G | G G | G G | C C |
| 11 | M | AD | G G | G G | A G | C A |
| 12 | F | AD | G A | G G | A G | C A |
| 13 | M | CTRL | G G | G G | A A | C A |
| 14 | M | CTRL | A A | C G | A G | C C |
| 15 | F | AD | G G | G G | A A | C C |
| 16 | F | AD | A A | G G | A G | A A |
| 17 | M | AD | G A | G G | A G | C C |
| 18 | M | AD | A A | G G | A G | C C |
| 19 | M | CTRL | G A | G G | G G | C A |
| 20 | M | CTRL | G A | C G | G G | A A |
| 21 | M | CTRL | G A | G G | G G | A A |
| 22 | F | CTRL | A A | C C | A G | C C |
| 23 | F | CTRL | G A | C G | G G | C A |
| 24 | F | CTRL | G A | G G | G G | A A |
| 25 | F | CTRL | A A | C G | A G | C A |
| 26 | F | AD | A A | G G | G G | C A |
| 27 | M | AD | G A | G G | G G | C A |
| 28 | M | AD | G G | G G | A G | C C |
| 29 | F | AD | G G | G G | A G | C C |
| 30 | F | AD | G G | G G | A G | C C |
| 31 | F | CTRL | G A | G G | G G | C C |
| 32 | M | CTRL | G A | C G | G G | A A |
| 33 | F | AD | G A | G G | A A | C A |
| 34 | M | CTRL | A A | C G | A G | C A |
| 35 | F | CTRL | G A | G G | G G | C A |
| 36 | F | CTRL | A A | C G | G G | C A |
| 37 | F | AD | G G | G G | G G | C A |
| 38 | M | CTRL | G A | C G | G G | A A |
| 39 | F | AD | G A | G G | A A | C C |
| 40 | M | AD | A A | G G | A A | C C |
| 41 | M | CTRL | A A | G G | G G | A A |
| 42 | F | CTRL | A A | G G | G G | C A |
| 43 | F | CTRL | A A | C G | G G | C A |
| 44 | F | CTRL | A A | C G | G G | C A |
| 45 | F | AD | A A | G G | A G | C C |
| 46 | F | CTRL | A A | C C | G G | C A |
| 47 | F | CTRL | A A | C G | G G | A A |
| 48 | F | CTRL | A A | C C | G G | C C |
| 49 | F | AD | A A | G G | A G | C A |
| 50 | F | AD | A A | G G | G G | C C |
| 51 | M | CTRL | G A | C G | G G | C A |
| 52 | M | AD | G A | G G | A G | C A |
| 53 | F | CTRL | A A | G G | A G | A A |
| 54 | F | CTRL | G A | G G | G G | C A |
| 55 | F | AD | G A | C G | G G | C C |
| 56 | M | CTRL | A A | C G | A A | C A |
| 57 | F | CTRL | G A | G G | G G | C C |
| 58 | M | AD | G A | C G | A G | C C |
| 59 | M | CTRL | A A | G G | A G | C A |
| 60 | F | AD | A A | G G | A G | C A |
| 61 | M | AD | G A | G G | G G | C C |
| 62 | F | CTRL | A A | C G | G G | C A |
| 63 | M | AD | G A | C G | A G | C C |
| 64 | M | AD | G A | G G | A A | C C |
| 65 | F | AD | G A | C G | A G | C A |
| 66 | M | CTRL | A A | G G | G G | A A |
| 67 | M | AD | A A | G G | G G | C C |
| 68 | F | CTRL | A A | G G | A G | C A |
| 69 | F | AD | G A | G G | A A | C A |
| 70 | F | CTRL | G A | G G | G G | A A |
| 71 | F | AD | A A | G G | A G | C C |
| 72 | M | CTRL | A A | G G | G G | C C |
| 73 | F | CTRL | A A | C G | G G | C C |
| 74 | F | AD | A A | G G | A G | C A |
| 75 | M | CTRL | A A | G G | G G | C C |
| 76 | F | CTRL | A A | C G | G G | C A |
| 77 | F | CTRL | G A | C G | G G | C A |
| 78 | F | CTRL | A A | G G | A A | C A |
| 79 | F | AD | A A | G G | A G | C C |
| 80 | F | AD | G G | G G | A G | C A |
| 81 | M | AD | A A | C G | G G | C C |
| 82 | F | AD | G G | G G | G G | A A |
| 83 | M | CTRL | A A | C C | G G | C A |
| 84 | M | CTRL | A A | C G | A G | A A |
| 85 | M | CTRL | A A | C G | G G | A A |
| 86 | F | AD | G A | G G | G G | C A |
| 87 | M | CTRL | A A | G G | G G | C C |
| 88 | M | CTRL | A A | C G | G G | C A |
| 89 | M | CTRL | A A | C G | G G | C C |
| 90 | F | CTRL | A A | C C | G G | C C |
| 91 | F | CTRL | A A | C C | G G | C A |
| 92 | M | CTRL | A A | G G | G G | A A |
| 93 | F | CTRL | G A | C G | G G | C A |
| 94 | M | CTRL | A A | G G | G G | C A |
| 95 | F | CTRL | G A | C G | G G | C A |
| 96 | F | CTRL | A A | G G | G G | C A |
| 97 | F | CTRL | A A | G G | A G | C A |
| 98 | M | CTRL | A A | C G | G G | C A |
| 99 | F | CTRL | G A | C C | G G | C A |
| 100 | F | CTRL | G A | G G | A G | C A |
| 101 | M | CTRL | A A | G G | G G | C A |
| 102 | M | CTRL | A A | G G | G G | A A |
| 103 | F | CTRL | A A | G G | G G | A A |
| 104 | F | CTRL | G A | G G | A A | C A |
| 105 | M | CTRL | G A | C G | G G | C A |
| 106 | F | CTRL | G A | C C | G G | C A |
| 107 | F | CTRL | A A | G G | A G | A A |
| 108 | F | CTRL | A A | G G | G G | C A |
| 109 | M | CTRL | A A | G G | A G | C A |
| 110 | F | AD | G A | C G | A G | C A |
| 111 | M | AD | G A | C G | A G | C A |
| 112 | F | AD | G A | G G | G G | C C |
| 113 | F | CTRL | G A | C C | A A | C C |
| 114 | F | AD | G A | G G | A A | A A |
| 115 | F | CTRL | A A | G G | G G | A A |
| 116 | F | CTRL | A A | C G | G G | A A |
| 117 | F | AD | A A | G G | A G | A A |
| 118 | M | AD | G A | C G | A A | C A |
| 119 | F | CTRL | G A | C C | G G | C C |
| 120 | M | CTRL | G A | C G | A G | A A |
| 121 | M | CTRL | A A | C G | G G | C C |
| 122 | M | CTRL | G A | G G | G G | C C |
| 123 | M | CTRL | A A | G G | G G | C C |
| 124 | F | AD | G A | G G | G G | C C |
| 125 | F | AD | G A | C G | G G | C A |
| 126 | F | AD | A A | C G | A G | C C |
| 127 | M | AD | A A | C G | A G | A A |
| 128 | F | AD | G A | G G | G G | A A |
| 129 | M | CTRL | G A | G G | A G | A A |
| 130 | F | AD | A A | G G | G G | A A |
| 131 | M | CTRL | G A | G G | A G | A A |
| 132 | M | CTRL | G A | G G | A A | C A |
| 133 | F | AD | A A | G G | A G | C C |
| 134 | M | CTRL | A A | C G | A A | C A |
| 135 | M | CTRL | A A | C G | A G | C A |
| 136 | F | CTRL | A A | C G | A G | A A |
| 137 | F | AD | A A | C G | A G | A A |
| 138 | F | CTRL | A A | C G | G G | C A |
| 139 | F | CTRL | A A | C G | G G | C A |
| 140 | F | CTRL | A A | G G | A G | C A |

Genotypes of the 140 Bearded Collies (55 AD cases, 85 healthy controls) analyzed at each of the four SNPs of interest: BICF2G630307993 on canine chromosome (CFA) 11, BICF2G630109748 on CFA16, BICF2P1230367 on CFA18 and BICF2P977298 on CFA29.

* M male; F female; AD Addison’s disease; CTRL healthy control
