# Supplementary material for: Genetic characterization of Addison’s disease in Bearded Collies
Source: BMC Genomics. 2020 Nov 26;21:833. doi: 10.1186/s12864-020-07243-0 (PMC7690126; doi:10.1186/s12864-020-07243-0)
Supplement: Supplementary file 3 — Additional file 3 : Table 2. Model assessment. Values for the leave-one-out information criterion (looic) and standard error associated with each of the predictive SNP models tested. The model with the smallest looic value is considered the best, most parsimonious, fit. [file 12864_2020_7243_MOESM3_ESM.docx]

**Additional Table 2. Model assessment**

| Model | SNP(s) | Looic value | Standard Error |
| --- | --- | --- | --- |
| Single SNP | CFA11 | 173.6 | 9.0 |
| Single SNP | CFA16 | 175.5 | 7.7 |
| Single SNP | CFA18 | 171.0 | 10.2 |
| Single SNP | CFA29 | 179.2 | 8.8 |
| 2-SNP | CFA11, CFA16 | 165.0 | 9.7 |
| 2-SNP | CFA11, CFA18 | 157.5 | 11.9 |
| 2-SNP | CFA11, CFA29 | 164.9 | 10.6 |
| 2-SNP | CFA16, CFA18 | 161.4 | 10.7 |
| 2-SNP | CFA16, CFA29 | 164.5 | 10.0 |
| 2-SNP | CFA18, CFA29 | 160.3 | 12.1 |
| 3-SNP | CFA11, CFA16, CFA18 | 151.9 | 12.1 |
| 3-SNP | CFA11, CFA16, CFA29 | 155.4 | 11.0 |
| 3-SNP | CFA11, CFA18, CFA29 | 147.5 | 12.7 |
| 3-SNP | CFA16, CFA18, CFA29 | 150.0 | 11.5 |
| 4-SNP | CFA11, CFA16, CFA18, CFA29 | 140.6 | 12.2 |

Values for the leave-one-out information criterion (looic) and standard error associated with each of the predictive SNP models tested. The model with the smallest looic value is considered the best, most parsimonious, fit.

* SNP single nucleotide polymorphism; CFA canine chromosome
