# Supplementary material for: Genetic characterization of Addison’s disease in Bearded Collies
Source: BMC Genomics. 2020 Nov 26;21:833. doi: 10.1186/s12864-020-07243-0 (PMC7690126; doi:10.1186/s12864-020-07243-0)
Supplement: Supplementary file 4 — Additional file 4 : Table 3. Haplotype blocks associated with Addison’s disease in Bearded Collies. Haplotype blocks associated with Addison’s disease on canine chromosomes 11, 16 and 18 in 103 unrelated Bearded Collies (41 cases, 62 controls). Locations represent the position of the first and last single nucleotide polymorphism (SNP) in each block and are based on the CanFam3.1 reference genome. [file 12864_2020_7243_MOESM4_ESM.docx]

**Additional Table 3.** **Haplotype blocks associated with Addison’s disease in Bearded Collies.**

| CFA | Haplotype block | Permuted  p-value | SNPs | CanFam3.1 location |
| --- | --- | --- | --- | --- |
| 11 | Block 1 | 0.0029 | TIGRP2P153246_rs8906130, BICF2P560672, BICF2P30914, BICF2P1088170, BICF2P1167407, BICF2P51703, BICF2P956000, TIGRP2P153295_rs9164620, BICF2P1396193, TIGRP2P153309_rs8755961, BICF2P1413358, TIGRP2P153317_rs8632754 | 69,206,106 – 69,322,515 |
|  | Block 2 | 0.0205 | BICF2P719729, BICF2P1247543, BICF2P73783, BICF2P855172, BICF2S23344916  TIGRP2P153419_rs8588049 | 69,531,917 – 69,597,920 |
|  | Block 3 | 0.0403 | BICF2G630307797, BICF2G630307811 | 71,207,600 – 71,215,715 |
|  | Block 4 | 0.0126 | BICF2G630307883, TIGRP2P153912_rs8552878, BICF2S2344488, BICF2G630307884, BICF2P292855, BICF2P798307 | 71,244,069 – 71,303,800 |
|  | Block 5 | 0.0178 | BICF2G630307924, BICF2G630307930, BICF2P1431974, BICF2P1254122, BICF2G630307966 | 71,323,983 – 71,364,581 |
|  | Block 6 | 0.0403 | BICF2S23334346, BICF2G630307972 | 71,408,253 – 71,411,126 |
|  | Block 7 | 0.0117 | TIGRP2P153986_rs9217231, BICF2G630307993*, BICF2P731554 | 71,436,665 – 71,456,789 |
| 16 | Block 1 | 0.0349 | BICF2P943539, BICF2P779044, BICF2S23322003, BICF2P589276, BICF2P160438, BICF2P1353251, BICF2P1101094, BICF2P1092354 | 24,603,563 – 24,745,324 |
|  | Block 2 | 0.0155 | BICF2P623442, BICF2P511602, BICF2G630109602, BICF2G630109609, BICF2G630109621, BICF2S2292173, BICF2G630109647, BICF2P926593, TIGRP2P213696_rs8835996 | 26,838,613 – 27,005,148 |
|  | Block 3 | 0.0155 | BICF2P1173477, BICF2P300470 | 27,013,605 – 27,016,595 |
|  | Block 4 | 0.007 | BICF2G630109701, BICF2G630109708, BICF2G630109723, BICF2G630109737, BICF2P103858, TIGRP2P213703_rs8513549, BICF2G630109748***** | 27,060,738 – 27,126,780 |
|  | Block 5 | 0.0181 | BICF2S23420465, BICF2P961638, BICF2G630109759, BICF2G630109763, BICF2P1165094, BICF2G630109802, BICF2G630109804 | 27,408,748 – 27,501,873 |
|  | Block 6 | 0.0231 | BICF2G630109820, BICF2P914129, BICF2S23160929, BICF2P1177601 | 27,520,193 – 27,545,515 |
| 18 | Block 1 | 0.0094 | BICF2S22952633, BICF2P954081, BICF2P404633, BICF2S23316104, BICF2P1040686 | 16,704,981 – 16,789,327 |
|  | Block 2 | 0.0201 | BICF2P163715, BICF2P1294812, BICF2P1392276, BICF2P993989, BICF2S23411890 | 27,438,390 – 27,502,704 |
|  | Block 3 | 0.0444 | BICF2P979307, TIGRP2P247286_rs9006990, TIGRP2P247299_rs9090020, TIGRP2P247303_rs8934970 | 27,603,545 – 27,642,904 |
|  | Block 4 | 0.0444 | BICF2P1071613, BICF2P1183510, BICF2P388124 | 27,916,624 – 27,931,371 |
|  | Block 5 | 0.0074 | BICF2S22929242, BICF2S22930123, BICF2S23022243, BICF2S2328153, TIGRP2P247524_rs8816334 | 28,555,191 – 28,614,375 |

Haplotype blocks associated with Addison’s disease on canine chromosomes (CFAs) 11, 16 and 18 in 103 unrelated Bearded Collies (41 cases, 62 controls). Locations represent the position of the first and last single nucleotide polymorphism (SNP) in each block and are based on the CanFam3.1 reference genome.

* Top GWAS SNP
